# A Framework for Clinicians to Improve the Decision-Making Process in Return to Sport

**DOI:** 10.1186/s40798-022-00440-z

**Published:** 2022-04-13

**Authors:** Kate K. Yung, Clare L. Ardern, Fabio R. Serpiello, Sam Robertson

**Affiliations:** 1 Institute for Health and Sport, Victoria University, Melbourne, Australia; 2 Musculoskeletal and Sports Injury Epidemiology Centre, Department of Health Promotion Science, Sophiahemmet University, Stockholm, Sweden; 3 Sport and Exercise Medicine Research Centre, La Trobe University, Melbourne, Australia; 4 Department of Family Practice, University of British Columbia, Vancouver, Canada

**Keywords:** Decision-making, Decision, Return to play, Decision analysis, Rehabilitation, RTS, RTP

## Abstract

Return-to-sport (RTS) decisions are critical to clinical sports medicine and are often characterised by uncertainties, such as re-injury risk, time pressure induced by competition schedule and social stress from coaches, families and supporters. RTS decisions have implications not only for the health and performance of an athlete, but also the sports organisation. RTS decision-making is a complex process, which relies on evaluating multiple biopsychosocial factors, and is influenced by contextual factors. In this narrative review, we outline how RTS decision-making of clinicians could be evaluated from a decision analysis perspective. To begin with, the RTS decision could be explained as a sequence of steps, with a decision basis as the core component. We first elucidate the methodological considerations in gathering information from RTS tests. Second, we identify how decision-making frameworks have evolved and adapt decision-making theories to the RTS context. Third, we discuss the preferences and perspectives of the athlete, performance coach and manager. We conclude by proposing a framework for clinicians to improve the quality of RTS decisions and make recommendations for daily practice and research.

## Key Points

- RTS decisions are complex, nonlinear and multifactorial and thus require external tools to assist practitioners
- To improve the quality of decisions in sports settings, decision-makers could evaluate the following three domains: (1) assess the methodological soundness of the tests chosen, (2) identify potential deviations from normative decision models and (3) implement shared decision-making.

## Introduction

Decision-making is a process of weighing the risk(s) and benefit(s) among options to make a choice [1]. In clinical practice, return-to-sport (RTS) decisions can be challenging as they are directly linked to the athlete’s well-being and performance. RTS refers to the recovery and rehabilitation continuum: return to participation, return to sport and return to performance [2]. This review focuses on how the quality of RTS decisions could improve.

Premature RTS may risk re-injury [3–5] and subsequently harm the athlete’s playing performance [6], financial income [7] and mental health [8, 9]. Yet, if RTS is delayed for a lesser chance of reinjury, it will inevitably reduce a team’s player availability. Lower player availability is undesirable as players’ match availability is associated with team performance across various sports [10–16]. Consequently, substantial pressure rests on the shoulders of decision-makers to reach a decision that balances the best interest of the athlete’s health and performance.

When the context is predictable and routine, for example when managing a tibia fracture on the field, decision-making could be straightforward and relegated to an automated level (i.e., remove from play immediately). However, when there is a high level of uncertainty and complexity in the context (e.g., to decide whether an athlete at 95% of recovery should play in the grand final), the ability to make high-quality decisions is less clear, yet potentially even more crucial.

The challenge of complexity and the multifactorial nature of RTS decision-making has been acknowledged for over two decades [17]. A 1998 review by Putukian [17] discussed the concerns and struggles that clinicians have when making RTS decisions, which could be attributed to the multifactorial nature and clinical uncertainty presented in medicine [18, 19]. The majority of the research focus since then has been mostly on developing decision-making frameworks and clinical criteria for RTS. One of the most recognised decision-making frameworks is the Strategic Assessment of Risk and Risk Tolerance (StAART) [20]. The framework, together with the RTS criteria, helps to guide a clinicians’ practice. For example, in the management of anterior cruciate ligament (ACL) injury, clinicians may refer to the established RTS criteria [21, 22] and consensus statements [23, 24].

In contrast to the vast literature on RTS criteria, there is less on how clinicians make RTS decisions and how to improve the quality of the decision. This may be because this topic spans at least two distinct fields: sports medicine and decision-making science. We aim to help clinicians conceptualise the decision-making process, increase the thoughtfulness of a decision, identify potential deviations from normative decision models and eventually establish a framework to improve the quality of decision-making.

### Disentangling Decisions and Outcomes

The term *decision* refers to the *action* taken to reach a decision, and this is different from the *outcome* of the decision [25, 26]. A high-quality decision refers to a decision that is logical and made based on the uncertainties, values and preferences of the decision-maker [27]. A good *outcome* is an outcome that the decision-maker would wish to have happened and is of high value to them [27].

A high-quality *decision* does not necessarily warrant a good *outcome* due to uncertainties. There are multiple sources of uncertainties, and the two major categories prominent in the medical field are aleatoric uncertainty and epistemic uncertainty [28]. Aleatoric uncertainty is intrinsic to the problem, for example, random variations that arise from observers or instruments. Epistemic uncertainty is extrinsic and comes from limitations in knowledge, such as individual bias [28].

Distinguishing between decision and outcome allows clinicians to separate action from the consequence, so they can focus on improving the quality of the action. Occasionally, clinicians may be disappointed by a bad outcome of a good RTS decision, such as an athlete suffering from a re-injury despite careful medical evaluation. Yet, in the pursuit of a good outcome, there may not be a better way than striving for a high-quality decision. Therefore, in this paper, we focus on evaluating the decision, and not on the outcome.

### Evaluating a Decision

There are various ways to evaluate a decision. The first approach is related to the *outcome* of the decision, such as clinical health outcomes (e.g., pain, quality of life), or how regretful or satisfied the patient is with the decision [29–31]. However, there is no consensus on the optimal measurement tool(s) for this purpose. The second approach relates to the *expected value* of the outcome (i.e., expected utility), where probabilistic information about the risk and benefits of personal preferences and values is considered [32]. The third approach is to consider the *decision quality*, which is measured by knowledge of the options and outcomes, realistic perceptions of outcome probabilities, and agreement between patients’ values and choices [29].

It may be challenging to measure the quality of a decision with the first two approaches (i.e., outcome and expected utility) due to the complexity of a RTS question. Nevertheless, it may be possible to evaluate the decision with the third approach—decision quality.

**Fig. 1:**
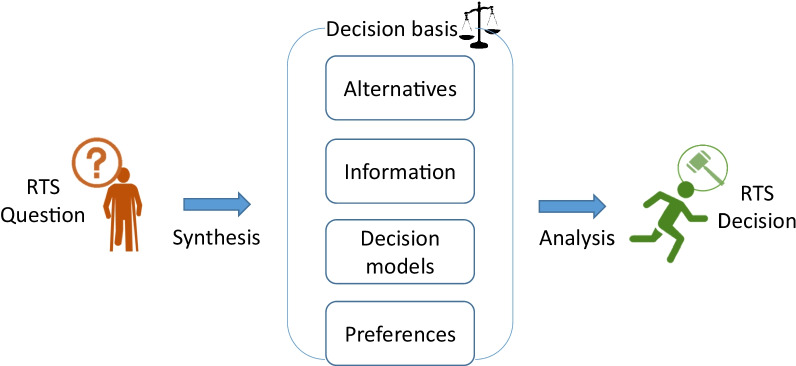
Steps for evaluating a RTS decision

Decision analysis is a formal procedure for analysing decision problems by balancing the factors that could influence a decision [27]. To evaluate the decision quality, the decision process could be made transparent by first breaking it down into a sequence of clear steps. We have adapted a decision analysis model from Howard [33] for RTS to systematically evaluate a decision (Fig. 1).

The essence of decision analysis is eliciting the four bases for the decision [33]:

1. *The alternatives* relates to the options that a decision-maker has. In the context of RTS after an injury, it could be whether the athlete could return to full training/competition, modified training or basic rehabilitation training.
2. *The information* refers to knowledge that may be important to formulate the outcome. For example, what information do RTS tests provide to the decision-makers?
3. *The decision models* include models that describe how the decision could be made. That is, on what basis can the decision be made?
4. *The preferences* of a decision-maker could be of multiple dimensions. These include the value (e.g., how much does RTS mean to the athlete or the team?), time preference (e.g., how important is it to play in the upcoming game?) and risk preference (e.g., how much re-injury risk can the team tolerate?).

Among the four key bases for a decision (alternatives, information, decision models and preferences), the alternatives are highly specific to the context and would be difficult to discuss from a broader perspective. Therefore, we have structured this review around the other three bases for a RTS decision: (1) information, (2) decision models and (3) preferences. We first zoom in to the methodological issues of obtaining *information* in the medical room. Second, we zoom out to identify the *decision models* relevant to RTS. Third, we discuss how *preferences* can be addressed with shared decision-making. Finally, we propose a framework to improve RTS decision-making in practice.

To increase the practicability of the framework and to help readers navigate the three bases for the RTS decision, a case scenario describing an ACL injury is used. We use ACL injury because it is a serious injury in sports that may threaten the career of athletes [6, 34]. Multiple clinical and performance tests have been developed to evaluate the readiness of the RTS [35], yet the re-injury risk of ACL remains high [36, 37] and some athletes do not return to sports following the injury [38].

## Part 1: Methodological Concerns in Information Gathering

*A football player, in her early career, has undergone an ACL reconstruction surgery six months ago and is eager to return to play. She wants to play as soon as possible to gain a contract extension but is also worried about getting reinjured. In the medical room, you sit with the player and decide on what kind of test to perform on-field and off-field.*

At the operational level, there are methodological considerations when gathering information for the decision. Below we discuss some of the underlying assumptions and methods concerns.

### Number of Criteria Used in RTS

In general, criteria-based RTS (e.g., muscle strength, functional and dynamic stability, and range of motion) have been suggested over a time-frame approach, which is to decide solely based on the athlete’s time spent in rehabilitation [39–43]. The ideal number of tests to use for this purpose may vary between cases. There are concerns that an insufficient number of tests may jeopardise the clinician’s ability to see the complete profile of an injured athlete. However, too many tests may increase the inherent error (e.g., athlete exhibiting reduced performance due to fatigue or reduced motivation) and exhaust more resources (e.g., staff, time, equipment). Currently, there is no recommendation for the ideal combination and number of tests to provide the most insight into the athlete’s readiness for RTS.

### Baseline Setting in RTS

Returning to pre-injury levels of health and fitness is often seen as the goal of RTS [2]. Therefore, setting an appropriate baseline provides an ideal foundation for clinicians to monitor progress by comparing the current functional and physical capacity of the athlete with previous preinjury data. However, it is challenging to set a baseline that is objective, replicable and suitable for the setting. For example, currently, there is no guideline on the timing and frequency for performing baseline tests. Adding more complexity to the problem, physiological and performance profiles often fluctuate daily due to periodisation in training and competition schedule (e.g., heart rate variability [44], musculoskeletal screening scores [45], hip strength and flexibility [46] and power (as in countermovement jump) [44].)

Here we used the limb symmetry index (LSI) as an example to illustrate the concerns with baseline setting. LSI is often included in the RTS protocol for ACL injury [22, 47–50]. LSI compares the performance of the involved limb with the uninvolved limb [51]. Often, a 90% side-to-side difference threshold is used as a passing score for RTS [47–50]. However, there is little scientific evidence on the optimal threshold. Even when limb symmetry is achieved, it does not necessarily indicate the athlete has reached a level sufficient for safe sports participation and performance [50, 52]. It is also questionable whether the uninvolved side could be used as the benchmark when pre-injury data are not available. After ACL reconstruction surgery, patients have reduced single-leg hop performance of both the involved and uninvolved sides [52, 53] and for up to 2 years after surgery [54]. This could be attributed to a combination of factors, such as deconditioning, fear or lack of motivation [54]. Consequently, defining the baseline measure for comparison remains a challenge and a suite of RTS tests have been recommended [2].

### Validity of RTS Tests

#### Content Validity

Content validity refers to how well a test protocol reflects what it intends to measure [55, 56]. Selecting measurement tools is important as unnecessary noise may dampen the accuracy of the decision model. If the tests selected are prone to false positives, clinicians may be unnecessarily delaying the rehabilitation process of the athlete [47].

Traditionally, in RTS decisions, clinicians would consider internal athlete data (e.g., physical fitness, strength, well-being, periodic health-screening, body-mass, anthropometric, internal load responses) and external factors such as training loads (e.g., running performance, training and match exposure), the timing in the season, and the importance of the game or training. However, there seems to be a bias towards assessing variables that are easily measured, and missing measures that may be important, but more difficult to measure [57]. For example, in the rehabilitation of an ACL injury, a clinician may assess the hip, knee and ankle joint alignment in jump and land testing to identify the extent of valgus or varus movement. The assessment may provide valuable information regarding movement strategies and physical capabilities of the athlete; however, it may not provide sufficient information regarding the performance in competition. In competition, an athlete may encounter different chaotic and unpredictable scenarios, such as unplanned movement tasks and under high opponent pressure and cognitive load. Despite the best intentions to design testing to be sports specific, the overall physical, psychological and emotional demands of a competitive match could be hard to replicate. Consequently, decision-makers may need to identify the content validity of the test and decide to interpret the test result.

#### Predictive Validity

Predictive validity is how well a test predicts performance on a criterion that is administered at a later date, such as RTS outcome [56, 58]. Predictive validity is only available for some of the tests such as hop tests [47, 59], single-leg bridge test [60] and psychological readiness test [61]. For most RTS tests, clinicians may not know whether passing the test means the athlete could achieve a satisfactory RTS outcome or not. In a recent study, there was no association between the predetermined functional performance test cut-offs and the risk of a new ACL injury [62]. Similar, the Landing Error Scoring System may not predict the ACL injury risk in a cohort of high school and college athletes [63].

### Responsiveness of RTS Test

Responsiveness, or sensitivity, refers to how well a test can detect meaningful changes in skill and functional assessment [55]. While it is important to track progress, recent evidence suggested that some common clinical tests cannot accurately track meaningful gains in biological and functional recovery after injury [64–66]. The time to normalise also differs. For example, in lower-limb injury assessment, 6-m timed hop test returned to normal earlier than the other three single-leg hop tests (single hop for distance, triple hop for distance and crossover hop for distance) [47]. Similarly, in hamstrings strain rehabilitation, straight leg raise returned to full at an early stage as compared to maximum hip flexion with active knee extension [64]. Limited literature is available to inform what tests are most suitable for informing treatment progression and rehabilitation progression [64].

### Meaningful Change in RTS Test Result

One of the purposes of conducting RTS tests is to assess the progression made in rehabilitation and to inform the RTS decision [2]. Statistical tests could identify whether the observed change in a particular RTS is due to true difference or the result of chance. The statistical tests, however, in isolation cannot indicate whether the change was clinically meaningful or could be reliably distinguished from random error in the measurement [67]. As such, there is a concept of “clinical significance” to describe whether the change is both noticeable and meaningful to the injured athlete. The clinically important difference refers to the difference in an outcome measure that is clinically meaningful [68]. For example, the smallest change required to detect a meaningful change beyond typical error for 6-m timed hop test is 12.96% [69]. For RTS tests where the data for meaningful change are unavailable, longitudinal tracking may help to identify a trajectory for an informed decision [47].

### Unknown Interaction Between Variables

In decision-making, there may be some pieces of information missing, whether known or unknown. For example, little is known about the linearity of soft tissue healing [70] or how compensation movement makes up quantitative symmetry (e.g., reaction and response time). There are also variables that a clinician may have not measured (e.g., knee movement in the worst chaotic scenario) or could not be measured (e.g., knee movement in an unplanned body contact or under extreme fatigue). The lack of measurement of cognitive load and sports-specific stimulus in rehabilitation may also expose a potential flaw in RTS decision-making [57].

## Part 2: Zoom Out to Identify the Decision-Making Framework and Theories

*You have gathered the information required and are deciding your stance on whether the athlete is suitable to return to play*.

**Fig. 2:**
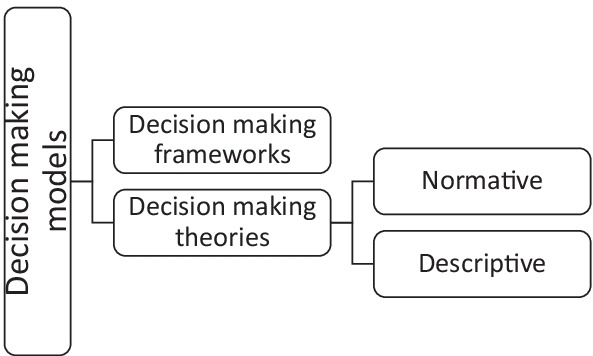
Overview of decision frameworks and theories

After gathering the information, here we zoom out to a broader perspective on decision-making models relevant to RTS. We first discuss a conventional RTS decision-making framework, then introduce the normative and descriptive decision models (Fig. 2). This allows clinicians an opportunity to see how a fully rational person may decide (normative models) and to explain when the decision could deviate from the norm (i.e., descriptive models).

### RTS Decision-Making Frameworks

In 2010, Clover and Wall [71] introduced a guideline for RTS decision-making. They proposed considerations for clinical factors and functional athletic ability. Intangible factors for RTS are also included, such as motivation of the athlete, social support, psychological readiness, fear of reinjury, insurance coverage and availability of rehabilitation team [71].

The first formal RTS decision-making guiding framework, a 3-step decision-based model, was proposed by Creighton et al. in 2010 [72]. The framework was designed to guide decisions on when to clear an athlete for full participation in sport without restriction. In 2015, minor revisions were made to the 3-step framework and it was renamed the Strategic Assessment of Risk and Risk Tolerance (StARRT) [20].

The StARRT has been used to clarify the components within and the sequence of decision-making and could help to explain the hidden assumptions that clinicians make in different clinical vignettes.

The process has three steps [72]:

- **Step 1** Evaluating health status. The health status of the athlete is evaluated through medical factors, such as symptoms, medical history, clinical objective tests and severity of the injury.
- **Step 2** Evaluating participation risk. The risk of participation is evaluated through the sport risk modifiers, such as the type of injury or illness, age, types of sports, level of play, the significance of upcoming competition, social factors and financial cost.
- **Step 3** Risk tolerance modifiers. The final step to RTS decision is a risk–benefit assessment by assessing the risk tolerance modifiers. These modifiers can exist at multiple levels (e.g., individual, interpersonal, organisational, community and policy levels) and may shift the decision-makers’ priorities and preferences. As a result, RTS decision-making could be more complicated than just a medical case.

The framework has helped make the decision-making process transparent by guiding the key variables that the clinician could consider [73]. However, the StARRT does not intend to define or guide a high-quality decision-making process. In the next section, various decision-making theories are introduced in an attempt to explain the decision-making process. Examples are provided to illustrate some of the methods by which a RTS decision could be reached.

### Decision-Making Theories

In decision-making, normative models and descriptive models form the two fundamental branches of decision theory [74]. Normative models are the system of rules and standards for decision-making (i.e., how one should make decisions). They have theoretical value and concerns about how to make the best possible decision when a person is fully rational and informed [74].

In contrast, descriptive models are psychological theory that explains how people actually make judgements and decisions [75]. Due to human behaviour, conflict occurs between how we would like to reason (normative) and our temptation (descriptive) of taking a faster or easier route in cognitive thinking. Descriptive models attempt to understand and explain the deviations from normative models. Here we use an example to illustrate the difference between normative and descriptive approaches: an athlete with an injury may know that alcohol could dampen recovery (a normative model explains what the athlete should do). Despite this, the athlete may still choose to drink at a party due to various reasons (a descriptive model explains why the athlete behaviour deviated from the normative model).

By comparing descriptive models to normative models, decision-makers may identify the potential deviations from normative models and correct the deviations if necessary. The section starts with normative models and is followed by descriptive models.

#### Normative Models

Common normative models include rule-based theory and explicit utility theory.

##### Rule-Based Theory

The rule-based approach is where a clinician decides based on a set of defined criteria [21, 22]. The assessment could be done on a binary scale (i.e., pass or fail). Table 1 illustrates a hypothetical example using established criteria for ACL injury [22]. Here we assume the relative importance and value assigned for all attributes are the same. The set of criteria includes seven tests, incorporating both function and subjective outcomes to reflect the overall knee performance. The passing criterion for RTS is to score > 90% on the seven tests [22].

**Table 1:**
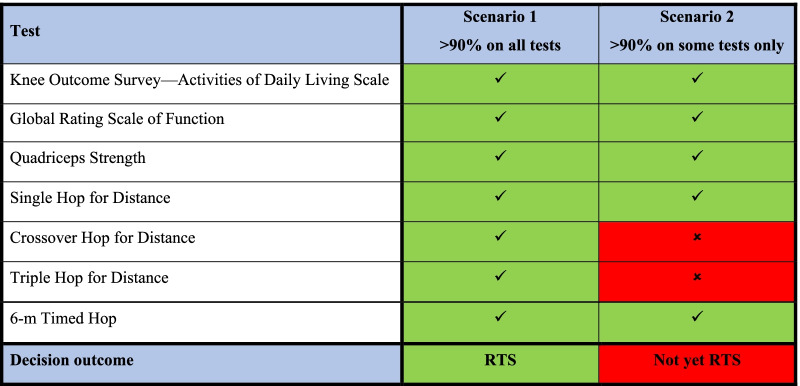
Hypothetical example of RTS criteria assessment, with criteria based on Grindem et al. [22] A tick suggests that the athlete has scored > 90% on that test, while a cross represents < 90%

###### *Example*

In scenario 1, the athlete scored above 90% on all tests below and is cleared to RTS. In scenario 2, not all tests are passed and the athlete is not cleared to RTS (see Table 1).

##### Expected Utility Theory

Expected utility theory is a decision model that illustrates how one decides in uncertain conditions, based on the outcomes of different options and the probability of each outcome [76, 77]. It assumes the decision made is rational as it is based on an assessment of the cost and benefit surrounding choices [78, 79]. Under this theory, a clinician makes a decision based on the utility (a subjective value assigned by the decision-makers) of the outcomes of different options and the probability (estimated likelihood) of each outcome [76, 77]. As with other normative models, expected utility theory assumes that decision-makers are fully rational in decision-making and have access to complete information about probabilities and consequences, in terms of time, resources and knowledge [20]. Table 2 shows a hypothetical calculation of weight utility value according to the same ACL RTS guideline as above [22].

**Table 2:**
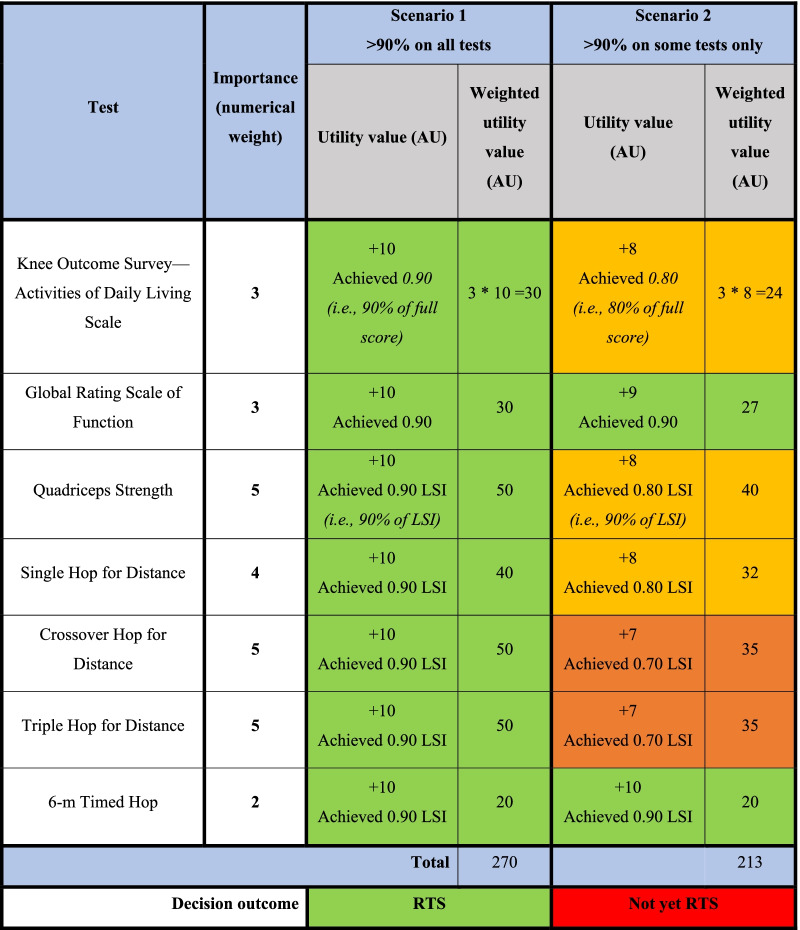
Hypothetical calculation using arbitrary units and utility value in ACL RTS, with criteria based on Grindem et al. [22]. Limb symmetry index (LSI)

###### *Example*

In Table 2, importance reflects how much the clinician values a specific test, and this is represented by a numerical weight. Utility value is based on the performance of the test, with 10 the highest score possible and 0 the lowest. In this case, achieving the goal of 90% LSI would correspond to a score of 10. The weight utility value is calculated by multiplying importance (numerical weight) by utility value (AU). For example, an importance of 3 and a utility value of 10 AU will give a weighted utility value of 30 AU (3 × 10AU = 30 AU). The highest possible weighted utility value in this example is 270AU and the decision is made based on the sum of the weighted utility value [80].

In scenario 1, the athlete achieved 90% on all the tests (indicated as “achieved 0.90”) and the sum of the weighted utility value is 270AU. The decision is RTS. In scenario 2, some of the tests have not passed the 90% threshold and the sum of the weighted utility value is 213AU. The weighted utility value has not reached the requirement set by the clinician, and the athlete was not cleared to RTS in scenario 2.

#### Descriptive Models 

Because humans are unlikely to be perfectly rational at all times, decisions made could deviate from a normative model. Systematic deviations from normative models are known as biases [75]. By applying normative models to the decisions made, decision-makers could look for possible biases and understand the nature of those biases with descriptive models. Examples of descriptive models include prospect theory, heuristics and bounded rationality [74]. With a better understanding of the biases, decision-makers could develop approaches to correct them (de-bias) and improve the quality of the decisions. The following section describes the common descriptive theories and how a decision may stray from the previous normative models.

##### Prospect Theory

Prospect theory suggested that people consider expected utility relative to a reference point rather than the absolute outcome. It also suggested that future gains and losses are asymmetrical, with losses having a greater emotional impact than gains (i.e., humans dislike losses more than potential gains).

###### *Example*

 In Table 2, the prospect theory would suggest that the decision-maker does not necessarily make decisions based on the absolute weight utility (i.e., 270AU). Instead, they would look at how far the expected utility is relative to a reference point (which is unknown here). If we adopt prospect theory in the context of RTS, a re-injury (loss) may bring a more negative emotional impact than winning (gain). While this may not be true in all cases, it may be worth noting the potential deviations in decision-making due to emotional distress.

##### Bounded Rationality

Bounded rationality describes how humans take reasoning shortcuts and make decisions within the bounds imposed by the environment, ability, information and goal [81]. The decision is rational; however, it is within the limits of information available to the decision-maker. That is, due to the limitation in accessing information, people tend to make sufficient judgements, rather than optimal ones [82, 83]. (For more details, see Gigerenzer and Goldstein [81] and Robertson and Joyce [83].) In RTS, not all meaningful data are collected due to various reasons, such as high cost, a lack of feasibility and time. Therefore, the best outcome for a decision made with unknown factors is not the same as decisions made in the context of transparency [84].

###### *Example*

 In the rehabilitation of an ACL injury, some information will always be unknown due to factors such as limitations in resources. This includes how we can accurately assess the degree of healing of the ACL graft after a reconstruction surgery or measure the loading capacity of the ACL. Consequently, the decision made by the clinician in the vignette is only based on the information available in Tables 1 and 2 and is limited by the cognitive capacity, and the knowledge and choices of the decision-maker.

##### Heuristic

Also known as a cognitive short cut, a heuristic is a decision-making strategy to act more quickly or frugally by ignoring parts of the information [85]. Heuristics allow people to make a rapid, efficient judgement without consuming a substantial amount of time, processing capacity, or when information is incomplete.

Logically, a clinician’s decision for RTS would be grounded in a more rational choice as described in normative models due to availability of time and opportunity to gather additional information from test or other staff members (e.g., doctors, coaches, fitness coach). However, RTS decision-making can also be based on heuristic decision-making, as seen when athletes make decisions regarding RTS [86].

There are many types of heuristics that are used in daily life [87]. Tversky and Kahneman [88] proposed three classes of heuristics which people may rely on to assess the probabilities of an uncertain event: availability heuristic, representativeness heuristic and anchoring and adjustment heuristic. In Table 3, we have suggested examples of heuristics that may be of relevance in RTS decisions. Heuristics sometimes may be useful in reducing the complexity of a task in assessing probabilities; however, it may also lead to systematic errors [88].

**Table 3:** Definitions and examples of heuristics in RTS

Heuristics	Definition	Example	Possible deviations from normative model
Availability	People infer the probability of an outcome based on how readily it comes to mind [88].	A clinician assesses the risk of injury of an athlete by recalling the recent occurrences within the team.	1. Depending on whether the clinician is familiar with the injury and when it last occurred, there may be recall bias. 2. The subjective probability of an injury may rise temporarily when the clinician sees there are multiple players on the injured list.
Representativeness	People categorise by matching the similarity of an object or incident to an existing one [88].	A clinician has an impression that a female athlete demonstrating knee valgus movement on a jump and land task will sustain a lower limb injury.	Evidence for screening tests in predicting injury is limited [89]. The clinician judgement may be insensitive to the reliability and predictability of the test.
Anchoring-adjustment	People estimate based on an initial value (anchoring) and adjust to yield the final answer (adjustment) [88].	A clinician prioritises information that supports his or her initial judgement of the estimated time to RTS and makes adjustments based on the initial value.	A clinician may stick to the initial hypothesis of the days required for RTS even if new evidence suggested conflicting information. Even if the clinician decides to adjust the estimation, it would be biased toward the initial value.

## Part 3: Preferences of the Decision-Makers

*You have consolidated the information and have weighed the risk and benefits of the medical clearance. Understanding that you are bounded by the information and knowledge available, you have used the rule-based theory described in Table* 1*as the basis for decision-making. Based on scenario 1, where the player has passed all of the tests, you have decided that the player is clinically fit to return to full training. Using the StARRT framework as a reference, you would like to discuss your rationale and other contextual factors with the athlete, coach and manager, to reach a shared decision.*

The StARRT framework helps clinicians make RTS decisions based on whether the risk assessment outcome exceeds the decision-maker’s risk tolerance[20]. That is, if the risk assessment is lower than the risk tolerance after all factors are considered, the athlete may be cleared to RTS. However, a *low* risk decision may not be synonymous with a *high-quality* decision.

In general medicine, it is recommended that the decision made by the clinician reflects the preferences of a well-informed patient, with consideration of factual and probabilistic health information [32, 90, 91]. There are multiple dimensions to address, including characteristics of the decision, knowledge and expectations of the situation and treatment options and outcomes, personal values and preferences, support and resources needed, personal characteristics and clinical characteristics [29, 91–93].

Practically, there is no optimal measurement tool that can measure the quality of the RTS decision based on the performance outcome or the expected utility of the decision-makers. However, a clinician can improve the decision quality by ensuring the decisions are well-informed and grounded in a shared decision-making approach.

### Improving Decision Quality by Shared Decision-Making

Shared decision-making has been a best practice for decision-making in the field of medicine [2, 94, 95]. It respects multiple perspectives and also aims to minimise disagreement due to conflicting interests.

Two phases characterise shared decision-making: 1) *deliberation* (pre-decisional, the process leading to a decision) and 2) *determination* (the act of decision) [96]. Deliberation is where knowledge is searched for, gained and appraised. To improve the quality of the shared decision, both the deliberation and determination could be evaluated [96]. An accurate judgment requires stakeholders to first collaborate to decide on the definition of success [2, 97]. Then decide on which pieces of information to pay attention to, nominate weighting and integrate the information [98]. This information may include the alternatives available, the advantages and disadvantages of the alternatives, the nature of the decision, the associated outcome and its likelihood [94, 96].

**Fig. 3:**
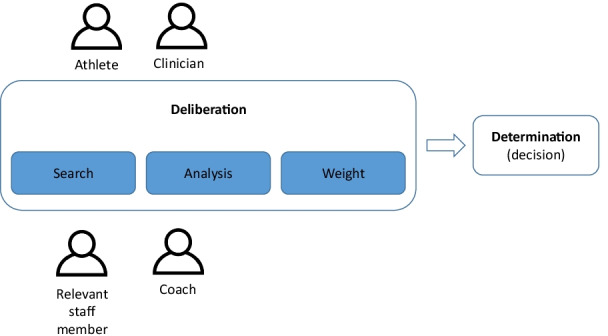
Shared decision model in sports. Adapted to RTS context from Elwyn et al.[94]

The second phase, determination, is to choose one of the options [96]. The actual decision may occur in a ‘black box’, where one combines the available information in their own way without transparency or accountability [99]. The lens decides how one interprets the “real” probabilities, which could be obscured by one’s cognitive and emotional influence. For example, how an athlete weighs the importance of his or her sports career may affect how the information is processed (Fig. 3).

Understanding the decision-making theories may allow decision-makers to realise the normative approach and thus engage in a high-quality and rational discussion during deliberation.

### The Perspectives of Decision-Makers

The keys to high-quality decision-making include accounting for individual preferences, social and contextual factors (e.g., the type of injury or illness, age, types of sports, level of play, the significance of upcoming competition and social factors and financial cost) [2, 32, 100, 101]. Social and contextual factors also impose constraints at multiple levels and influence the RTS decision, including at individual, interpersonal, organisational, community and policy levels [72, 73, 102]. The factors may shift the athlete’s and decision-makers’ priorities and preferences, which make decision-making more complicated [20, 72].

Traditionally, clinicians are the gatekeeper of the RTS decision [71, 103–107]. The clinician has skills in assessing the injury-related criteria in RTS, including assessing the state of healing, risk of re-injury and risk of short- or long-term problems [96, 104, 108, 109]. Clinicians also have an overriding duty of care to patients and a legal and ethical obligation to act in a manner that is necessary and appropriate to protect the health of an athlete.

However, with the addition of trainers, rehabilitation coaches and performance coaches, clinicians are no longer the only staff contributing to rehabilitation and RTS decisions. It is questionable whether clinicians should still be the main advisor for RTS decisions, given the numerous non-medical factors to consider [97, 100, 103, 108, 110–113]. In a sports setting, a clinician may even have dual allegiances, as the clinician does not work exclusively for the patient, but also on behalf of the club or organisation. They may experience pressure from their employer (i.e., the sports organisation) to minimise lay-off time and to clear an athlete as soon as possible. As such, an inherent conflict of interest may present in a professional sports team setting [114, 115].

The following section discusses the general concerns and considerations of the athlete and coaches to improve communication transparency and to minimise conflicts.

#### Athlete

There are internal and external factors influencing how an athlete may view the quality of the decision and listening to their opinions may be beneficial to inform the final decision [101]. Internal factors include perception of body, self-resentment [116, 117] and their emotional tie to their sport [117]. External factors include sociocultural influences, such as financial concerns, expectations from family and friends and their given sport’s culture of risk [118, 119]. Some athletes may face social pressure to perform [118]. Social pressure could be the pressure to meet the expectations of peers, fans and coaches [116, 117, 120–122]. Shame and alienation from the team due to injury may lead to low self-esteem and depression [120, 122, 123].

There is limited evidence on how athletes approach decisions about RTS, especially in a complex and risky scenario. ‘Playing hurt’ is a common phenomenon across different sports, age groups and performance levels [117–119, 124, 125]. However, it is unclear how and when an athlete would choose to play hurt.

In a recent study that investigated how athletes decide on RTS [86], athletes would consider the relevance of the competition (e.g., the importance of the competition), potential sporting consequences (e.g., loss of the starting position) and whether the risk of playing hurt could be offset by some means (e.g., availability of protective gear or possibility of being removed from play if pain increases). If the medically safe alternative (e.g., withdrawal from competition) does not have severe sporting consequences (e.g., loss of starting position), the athlete may opt for it. In contrast, if playing hurt may produce a sporting consequence that the athlete cannot afford but the risk of playing could be subjectively reduced, they may choose to play hurt. Clinicians and coaches can be influential in the athlete’s decision-making as clinicians and coaches are likely to know about the sporting consequences and the possibility of risk reduction.

As opposed to the risk analysis suggested in the normative StARRT framework [20], not all athletes attempt to obtain information actively and comprehensively [86]. Therefore, it may be helpful for clinicians and coaches to guide athletes through the information seeking process and provide a full picture of the situation and the sporting consequence.

#### Performance Coach and Manager

In some settings, coaches and managers could be the decision-makers for RTS, and thus, it is important to have their perspective as well. Coaches and managers are competent in assessing the non-injury-related RTS criteria, such as the athlete’s desire to compete, psychological impact, financial consideration and loss of competitive standing [108].

Based on existing literature, some coaches believe they have a responsibility to push the athlete to their limits, mentally and physically to achieve excellence in performance [126]. While some coaches act according to the training restriction implemented to reduce injury risk [122], some perceive prolonged or delayed RTS as harmful to the overall and long-term performance of the athlete [122]. Some coaches also believe clinicians are overly cautious and delay RTS of athletes unnecessarily [122]. However, research is scarce and based on small sample size, thus limiting generalisability.

To facilitate rehabilitation, coaches and managers may help to remove the barriers arising from the social and environmental context [127]. For example, they can ensure that athletes have sufficient resources to access adequate supervised rehabilitation. Coaches and managers can also ensure all relevant personnel are provided with information regarding the injury and the rehabilitation progression. These actions may increase transparency in communication and facilitate the decision to include or exclude from the main training group [127].

There are times when clinicians might miss something important without realising it. Shared decision-making may help to minimise the blind spots by filling the missing gaps and broadening the perspectives.

## Practical Implication

**Fig. 4:**
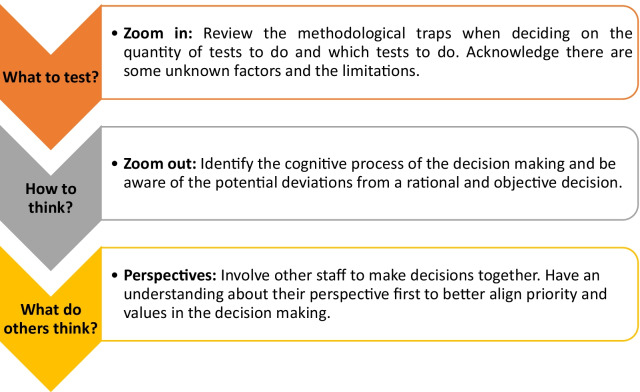
Three steps to making a high-quality RTS decision

Based on a decision analysis model, we have outlined a framework to help clinicians make systematic and objective RTS decisions. The first step is to choose appropriate RTS tests and to synthesise the information in a meaningful way. The second step is to understand the decision-making theories and identify possible deviations from normative models. The third step is using shared decision-making to improve decision quality by eliminating the contextual ‘blind spots’, such as an individual’s expectation, preference and value. We propose a framework that clinicians could refer to when they decide on RTS in a sports organisation (Fig. 4).

### Future Research

Currently, there is limited evidence or expert knowledge on how clinical decisions in sports are made, especially for upper-limb injuries. While in principle, the decision-making process of other sports injuries would be similar, future research could also investigate upper-limb injuries, for example, a shoulder dislocation injury. Similarly, there is little attention paid to how heuristics may be present in sports medicine practice. Research is needed to identify the heuristics used in clinical practice as limited work has been done in the field. Strategies for better judgment and decisions, such as reducing bias, are also required.

Another concern is the increasing number of data types with the growth of sports technology. There is a certain point where additional information no longer improves a human’s ability to make better decisions [128]. The human mind has an upper limit for information processing capacity and is sufficiency sensitive to large inconsistencies, but not small ones [129, 130]. Providing more information than the upper limit would only exhaust one’s cognitive information capacity in decision-making, potentially leading to overload, poor decision-making, and dysfunctional performance [131]. Consequently, there is an urge to identify tools that aid human brains in making decisions.

Examples of these decision-making tools could be statistics, mathematical modelling and artificial intelligence (AI) algorithms. In particular, machine learning techniques, a subfield of AI, attracted attention for their strength to transform a large amount of data into useful knowledge and identify nonlinear patterns [132–134]. In many cases, these external aids may complement or be superior to human performance [135–137]. Currently, the application of the above tools mostly remains on the theoretical level. Future research may explore how these tools may be applied on a practical level.

## Conclusion

The purpose of this review was to provide an overview of RTS decision frameworks and what constitutes high-quality decision-making. There is a lack of empirical knowledge in RTS decision-making and the potential adaptations within its process; most research focuses on biological and medical factors. One of the strengths of the review is to lay out the decision basis and hence the transparency of a decision. Understanding decision-making theories in the context of RTS and potential deviations from normative decisions may improve the work process and quality of decision-making. More research is required to understand how decisions are made and how to use computation tools to support and improve decision quality.

## Data Availability

Not applicable.

## Abbreviations

AI: Artificial intelligence
ACL: Anterior cruciate ligament
LSI: Limb symmetry index
RTS: Return-to-sport
StARRT: The Strategic Assessment of Risk and Risk Tolerance
